# A Multi-Scale Submodel Method for Fatigue Analysis of Braided Composite Structures

**DOI:** 10.3390/ma14154190

**Published:** 2021-07-27

**Authors:** Jincheng Zheng, Peiwei Zhang, Dahai Zhang, Dong Jiang

**Affiliations:** 1 School of Mechanical and Electronic Engineering, Nanjing Forestry University, Nanjing 210037, China; zhengjincheng@njfu.edu.cn; 2 Institute of Aerospace Machinery and Dynamics, Southeast University, Nanjing 211189, China; zhangpeiwei@seu.edu.cn (P.Z.); dzhang@seu.edu.cn (D.Z.)

**Keywords:** 2D braided CMCs, multi-scale fatigue life analysis method, sub-model, 2D SiC/SiC stiffened plates, random tension–tension loading

## Abstract

A multi-scale fatigue analysis method for braided ceramic matrix composites (CMCs) based on sub-models is developed in this paper. The finite element shape function is used as the interpolation function for transferring the displacement information between the macro-scale and meso-scale models. The fatigue failure criterion based on the shear lag theory is used to implement the coupling calculation of the meso-scale and micro-scale. Combining the meso-scale cell model and the fatigue failure criterion based on the shear lag theory, the fatigue life of 2D SiC/SiC is analyzed. The analysis results are in good agreement with the experimental results, which proves the accuracy of the meso-scale cell model and the fatigue life calculation method. A multi-scale sub-model fatigue analysis method is used to study the fatigue damage of 2D SiC/SiC stiffened plates under random tension–tension loads. The influence of the sub-models at different positions in the macro-model element on the analysis results was analyzed. The results shows that the fatigue analysis method proposed in this paper takes into account the damage condition of the meso-structured of composite material, and at the same time has high calculation efficiency, and has low requirements for modeling of the macro finite element model, which can be better applied to the fatigue analysis of CMCs structure.

## 1. Introduction

CMCs have the advantages of high-temperature resistance, high specific strength and specific modulus, etc., and are ideal materials for high-performance aircraft [1,2]. According to the meso-structure, CMCs can be classified into three types: unidirectional, laminated, and braided [3,4,5]. Braided CMCs have better mechanical properties than unidirectional and laminated CMCs, which overcomes the problem of low interlayer performance, easy delamination and cracking [6,7]. The braided CMCs are the key components of advanced aircraft [8]. The components of braided CMCs in aircraft often be subjected to fatigue load. Therefore, the fatigue analysis is the key problem in the application of braided CMCs.

The fatigue life analysis methods of CMCs mainly include the macroscopic phenomenological method and the method based on microscopic damage mechanism [9]. The macroscopic phenomenological method is to establish the macroscopic mechanical property decline model of CMCs, such as residual stiffness model and residual strength model, based on a large number of fatigue test data. Wu et al. [10] derived the residual stiffness model of composite materials under random loads based on the macroscopic phenomenological residual stiffness model of composite materials under constant amplitude loads, and determined the parameters in the model through experimental data. Fang et al. [11] proposed a two-dimensional residual strength model of needled CMCs considering wear and running-in mechanisms. The tensile fatigue tests and residual tensile strength tests of 2-D needled CMCs were carried out, and the relevant parameters in the residual strength model were identified by the date of tests. However, the macroscopic phenomenological method usually requires a lot of fatigue test data, which will take up high time cost and economic cost, and it is difficult to reflect the fatigue damage status of the meso-structure. The method based on microscopic damage mechanism is to establish the mechanical model of fatigue damage based on the meso-fatigue failure mode [12]. Min et al. [13,14] established a multi-scale fatigue calculation model based on micro mechanics for plain woven C/SiC CMCs, and verified the effectiveness of the proposed method by comparing it with fatigue test data of plain-woven C/SiC CMCs. Based on the fatigue hysteresis behavior of CMCs, Li et al. [15,16,17,18] established a fatigue life prediction model which considering the decline of interfacial shear stress and fiber strength during the fatigue cycle. The fatigue life S–N curves of unidirectional; laminated; and 2D, 2.5D, and 3D braided CMCs are predicted, which are in good agreement with the experimental results. However, it is difficult to establish the meso-mechanical analysis model, which requires the theoretical modeling of the complex meso-structure of braided CMCs. In recent years, due to the promotion of commercial finite element software [19], many researchers have developed the fatigue life calculation method of composite materials based on finite element model. Naderi et al. [20] established the macroscopic equivalent finite element model of carbon/epoxy resin laminated composites, and used Gaussian distribution to simulate the discrete type of material strength and stiffness parameters. The macro finite element model was used to analyze the fatigue life of carbon/epoxy resin laminated composites, and the analysis results were in good agreement with the test results. Yang et al. [21] established the meso-structure geometric model of 3D braided composites, studied the fatigue behavior under three-point sinusoidal waveform bending by means of tests and finite element analysis, and obtained the fatigue damage of yarn and matrix. The damage patterns of experiments and finite element analysis results were very consistent. Taking two-dimensional braided C/SiC composites as the research object, Wang et al. [22] established the meso-cell model and micro-cell model respectively, and fitted the fatigue damage evolution equation of fiber bundle by using the uniaxial tensile fatigue test data of two-dimensional braided C/SiC composites. The fatigue damage process of a unit cell model was analyzed under uniaxial, biaxial, and shear loads, and the relationship between the damage degree and the number of cycles in each direction was obtained. Equivalent stress equation and anisotropic macroscopic damage evolution equation of plane braided C/SiC composites are established. Finally, the equivalent stress formula and anisotropic macroscopic damage evolution equation are used to estimate the random vibration fatigue life of C/SiC composite plane braided plate, which is in good agreement with the experimental results. The fatigue life analysis method based on finite element can avoid the complicated meso-geometric theory modeling. However, the above methods are mainly applied to the fatigue life analysis of material scale, and are difficult to be directly applied to the fatigue life analysis of ceramic matrix composite structures.

The fatigue life analysis of composite material structure first needs to obtain the stress state of the fatigue hot spot of the structure under the action of fatigue load [23]. Since the braided CMCs material has obvious structural characteristics, it has three scales: micro-scales, meso-scales, and macro-scales. When performing finite element fatigue analysis on the CMCs structure, the macroscopic large-scale grid finite element model modeling and calculation efficiency High, but the calculation accuracy is difficult to guarantee; the calculation accuracy of the fine model that can characterize the meso-structure is high, but the modeling difficulty is high and the calculation efficiency is low. In order to solve the problems of computational accuracy and efficiency caused by mesh size in the finite element modeling process, the local mesh refinement method [24,25], substructure method [26,27] and sub-model method [28,29] are mainly adopted at present. The local mesh refinement method adopts small size mesh in the key parts of the structure and large size mesh in other areas. However, for braided CMCs, due to the complex meso-structure shape and the large-scale difference between the meso-structure and the macro structure, the modeling of the transition region is too difficult and the mesh quality is difficult to guarantee. The substructure method can reduce the order of large composite structures by substructure division, polycondensation and modal synthesis, but the calculation accuracy of substructure method has a great relationship with the selection of polycondensation mode, residual structure and the number of polycondensation nodes, so it is difficult to guarantee the accuracy of calculation results [30]. Sub-model method cuts out the key areas of the structure from the overall structure as sub-models, performs refined modeling on the sub-models, and simultaneously solves the overall model and the sub-models. [31,32]. Jiang et al. [33] used a multi-scale method to study the damage and crack debonding of braided composite materials under impact load. A macro model and a meso model are established respectively. The microscopic failure conditions such as matrix cracking and fiber/matrix interface debonding are analyzed based on the meso model, and the macroscopic model of impact failure is analyzed based on the macro model. Daghia et al. [29] proposed a meso-micro-scale calculation method based on a discretization model and a continuity model. The coupling calculation between the macro- and meso-models is carried out by establishing the interface between the macro-model and the meso-model. The meso model can be calculated by the standard finite element method, and the micro model needs to be calculated by the dedicated LATIN method. However, this method requires that the boundary of the meso-model is consistent with the spatial position of the micro-model, which limits the application of this method. A multi-scale fatigue analysis method based on sub-model is proposed in this paper. With the finite element shape function as the interpolation function and the node displacement of the macro model as the boundary condition of the meso model, the coupling calculation of macro and meso model is realized.

Combined with the sub-model method and the CMCs fatigue failure criterion based on the shear lag theory, this paper implemented the coupling calculation of three scales: macroscopic, mesoscopic, and microscopic. The fatigue life calculation of the braided CMCs based on 2D SiC/SiC meso sub-model was carried out in this paper. Taking the stiffened plate structure as the research object [34], the fatigue life of 2D SiC/SiC cross stiffened plate under amplitude load is analyzed. The influence of the embedding position of different meso-models in the macro-model on the calculation results is then studied. The accuracy and applicability of the multi-scale fatigue life analysis method based on sub-model is also verified.

## 2. Multi-Scale Fatigue Analysis Method Based on Sub-Model

### 2.1. Fatigue Failure Criteria for CMCs

The fatigue failure modes of CMCs mainly include matrix cracking, interface debonding, fiber fracture, etc. [35]. When subjected to fatigue load, if the fatigue peak stress is greater than the matrix cracking stress, the initial crack will occur in the matrix and debonding will occur at the fiber/matrix interface. In the subsequent fatigue loading process, due to the continuous unloading and reloading, the fiber and matrix will slip and friction at the fiber/matrix interface, which will lead to the wear of the fiber and fiber/matrix interface. With the increase of the number of fatigue load cycles, fiber strength will decrease with the cycle due to fiber wear. At the same time, due to the wear of the interface, the ability of the interface to transfer load between the fiber and matrix will decrease, and the fiber will bear more load with the increase of the number of cycles. These two factors will cause the failure probability of fiber increased with the increase the number of cycles. Fatigue failure of composite material can be determined when the failure probability of fiber reaches critical value.

For 2-D braided CMCs, only consider the damage caused by the sliding friction of the fibers and matrix inside the yarn, and the damage caused by the sliding friction between the yarn and the yarn or between the yarn and the matrix outside the yarn is ignored. Therefore, the fatigue failure analysis of 2-D braided CMCs can be simplified to the fatigue analysis of yarn, and the yarn belongs to unidirectional CMCs in nature.

For unidirectional CMCs, it is assumed that when some fibers fail, the intact fibers and the broken fibers share the load, then the stress exerted on the composite distal end and the stress borne by the intact and broken fibers satisfy the following relationship [36]

(1) $$\frac{\sigma }{V_{f}}=T\left[1-P\right]+T_{b}P$$

where *V_f_* represents fiber volume fraction, σ represent the stress carried by composite material, *T* represent the stress carried by intact fibers, *T_b_* represents the stress carried by broken fiber. Then, the probability of fiber failure can be expressed as

(2) $$P=1-\exp\left[-{\left(\frac{T}{\sigma_{c}}\right)}^{m_{f}+1}\right]$$

where *σ_c_* represents characteristic fiber strength, *m_f_* represents the Weibull modulus of fiber.

The critical fraction of broken fibers is given as [37]

(3) $$P^{*}=2/\left(m_{f}+2\right)$$

Due to the slip and friction between the fiber and the fiber/matrix interface during the fatigue loading process, the interface will wear, and the load transfer capacity of the interface decreases with the loading cycles. Fibers will wear and tear, and fiber strength decreases as the loading cycles progresses. Evans et al. [38], proposed an empirical formula for the decay of the interface shear stress of CMCs with loading cycles

(4) $$\frac{\tau \left(N\right)-\tau \left(0\right)}{\tau_{\min}-\tau \left(0\right)}=1-\exp\left(-\omega N^{\lambda }\right)$$

where *τ_min_* represents the shear stress of the interface after wear to a stable state, *τ*(0)** is the initial interfacial shear stress, *τ*(*N*)** represents the interfacial shear stress at the cycle *N*,* ω*, *λ* are empirical parameters.

Lee et al. [39] proposed the empirical formula of fiber strength decline with cycle

(5) $$\frac{\sigma_{0}\left(N\right)}{\sigma_{0}}=1-P_{1}{\left(\log N\right)}^{P_{2}}$$

where *P*_1_ and *P*_2_ are empirical parameters.

The fiber failure probability of each loading cycle can be determined by substituting Equations (4) and (5) in Equation (2)

(6) $$P\left(N\right)=1-\exp\left[-{\left(\frac{T}{\sigma_{c}}\right)}^{m_{f}+1}{\left(\frac{\sigma_{0}}{\sigma_{0}\left(N\right)}\right)}^{m_{f}}\frac{\tau \left(0\right)}{\tau \left(N\right)}\right]$$

where *σ*_0_ represents fiber reference strength within the reference length *l*_0_, *σ*_0_(*N*) represents the fiber strength at the cycle *N*, *σ_c_* can be calculated from the tensile strength *σ_uts_*

(7) $$\sigma_{uts}=V_{f}\sigma_{c}{\left(\frac{2}{m_{f}+2}\right)}^{\frac{1}{m_{f}+1}}\left(\frac{m_{f}+1}{m_{f}+2}\right)$$

**Figure 1:**
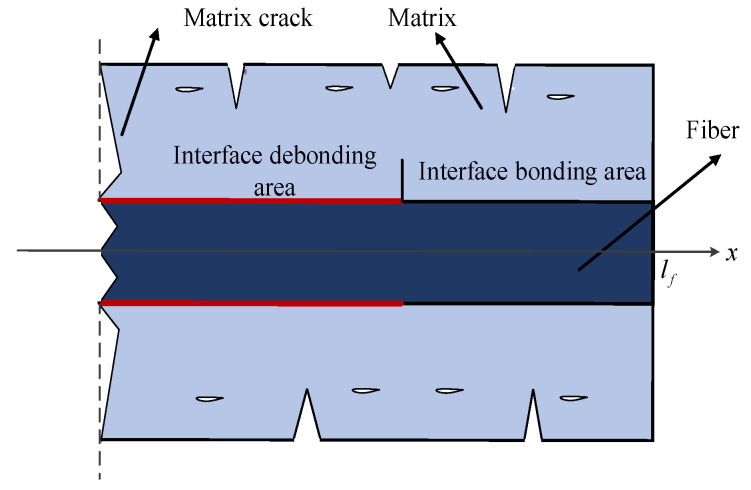
Fiber fracture area.

As shown in Figure 1, when the fiber is broken, the fiber/matrix interface is bonded in the area far away from the breaking point of the broken fiber, so the load can be transferred between the fiber and matrix through the interface. At the breaking point, the interface is unable to transfer load due to debonding. Assuming that the fiber does not bear load at the breaking point, the stress borne by the broken fiber can be expressed as

(8) $$T_{b}\left(x\right)=\frac{2\tau_{i}\left(N\right)}{r_{f}}x$$

After the fiber is broken, the load it bears will drop instantly. In order to restore the stress borne by the broken fiber to before the break, the broken fiber needs to continue sliding relative to the matrix. Suppose that the slip length required by the stress borne by the broken fiber to restore to its pre-fracture stress is

(9) $$l_{f}=\frac{r_{f}T}{2\tau \left(N\right)}$$

The probability density function *f(x)* of fiber fracture within the range ±*l_f_* from the matrix crack plane is [40]

(10) $$f\left(x\right)=\frac{1}{P\left(N\right)l_{f}}{\left(\frac{\sigma_{c}}{T}\right)}^{m_{f}+1}{\left(\frac{\sigma_{0}\left(N\right)}{\sigma_{0}}\right)}^{m_{f}}\frac{\tau \left(N\right)}{\tau \left(0\right)}P\left(N\right),x\in \left[0,l_{f}\right]$$

According to Equations (5)–(7), the average stress borne by the broken fiber can be obtained

(11) $$T_{b}=\int_{0}^{l_{f}}T_{b}\left(x\right)f\left(x\right)dx$$

Combined with Equations (1), (4)–(6), and (10), the relationship between stress exerted by composite materials, number of cycles and stress borne by intact and fractured fibers can be obtained [36]

(12) $$\sigma =V_{f}T{\left(\frac{\sigma_{c}}{T}\right)}^{m_{f}+1}{\left(\frac{\sigma_{0}\left(N\right)}{\sigma_{0}}\right)}^{m_{f}}\frac{\tau \left(N\right)}{\tau \left(0\right)}\left\{1-\exp\left[-{\left(\frac{T}{\sigma_{c}}\right)}^{m_{f}+1}{\left(\frac{\sigma_{0}}{\sigma_{0}\left(N\right)}\right)}^{m_{f}}\frac{\tau \left(0\right)}{\tau \left(N\right)}\right]\right\}$$

The fatigue failure judgment process of unidirectional CMCs is as follows: first, input the macroscopic overall fatigue peak stress *σ* and the number of cycles on the composites *N*, the stress *T* borne by the intact fiber can be solved by Equations (4), (5), and (12). Then put the stress *T* borne by the intact fiber into Equation (6) to calculate the volume fraction *p*(*N*)** of the failed fiber, when the volume fraction of the failed fiber reaches the critical value p*, the composite material fails; At this point, the number of cycles *N* is the fatigue life of the composite material under the fatigue peak stress.

### 2.2. Fatigue Damage Accumulation Theory

Miner believed that under constant amplitude fatigue load, the network absorbed by the material is equal with each cycle, the amount of damage to the material in each cycle was the same

(13) $$\frac{\Delta W}{W}=\frac{1}{N}$$

where *N* is the fatigue life of the material under the constant amplitude fatigue load

The dimensionless damage factor *d* was introduced. The amount of damage caused to the material by constant amplitude fatigue cycle load was related to the number of cycles *n* under this load and the fatigue life *N* corresponding to this fatigue load level [41]

(14) $$d=\frac{n}{N}$$

For multi-stage fatigue load, the total fatigue damage is linear accumulation of the fatigue damage caused by each stage. Define *N_i_* as the fatigue life at the fatigue load level of *σ_i_*, and *n_i_* is the number of cycles at this fatigue load level, then the damage quantity *D* can be expressed as

(15) $$D=\overset{N}{\underset{i=1}{\sum }}\frac{n_{i}}{N_{i}}$$

The fatigue failure of the composite material occurs when the total damage *D* = 1.

Assuming *t* is the multi-stage fatigue loading time, the fatigue life of the structure can be expressed as

(16) $$T=\frac{t}{D}$$

### 2.3. Sub-Model Method Based on Shape Function Interpolation

The principle of the multi-scale finite element analysis method based on sub-model is to obtain the stress and strain information at the meso-scale by constructing the multi-scale basis function for downscaling calculation on the basis of solving the response of the macro model [42]. This method can obtain more accurate analysis results when solving the macro-structure response. The sub-model method based on node displacement information transmission is adopted in this paper. Its principle is that the node displacement response results of the macro equivalent model are applied to the meso-fine model as boundary conditions, and then the high-precision response results are obtained by solving the meso-fine model.

Since the number of nodes on the boundary of meso-fine model is far more than the number of nodes on the boundary of the macro-model, when the macro-model transmits the node displacement information to the meso-fine model, some nodes on boundary of the meso-fine model cannot correctly match the displacement information, which will lead to inaccurate results. In this paper, the finite element shape function [43,44] is used as the displacement interpolation function, and the displacement response information of the nodes in the macro-equivalent model corresponds to the boundary nodes of the meso-fine model for interpolation operation.

**Figure 2:**
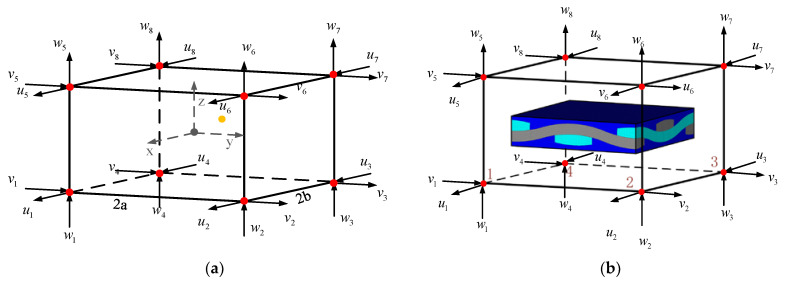
Interpolation based on finite element shape function. (**a**) Eight-node hexahedron element; (**b**) Obtaining the boundary conditions of the meso-scale model.

As shown in Figure 2a, an element of eight-node hexahedron is taken as an example, define the geometric center of the 6-hedron as the origin of the coordinate axes, and each side is parallel to the *X*, *Y*, and *Z* axes respectively. The side lengths of the hexahedron element are 2 *a*,** 2 *b*, and 2 *c* respectively. When the displacement of eight nodes along the directions *x*,* y*, and z is known to be *u_i_*,* v_i_, w_i_*. If the displacement of 8 nodes along the three directions *x*,* y*, and *z* is known to be *u_i_*,* v_i_*,* w_i_*, then any point *p* inside the hexahedral unit, whose coordinate is *(x*,* y*,* z)*, its displacement component *u*,* v*,* w* along the three directions *x*,* y*, and *z* are trilinear, which can be expressed as

(17) $$\begin{array}{cc} u & \;=d_{1}+d_{2}x+d_{3}y+d_{4}z+d_{5}xy+d_{6}xz+d_{7}yz+d_{8}xyz\\ v & =d_{9}+d_{10}x+d_{11}y+d_{12}z+d_{13}xy+d_{14}xz+d_{15}yz+d_{16}xyz\\ w & =d_{17}+d_{18}x+d_{19}y+d_{20}z+d_{21}xy+d_{22}xz+d_{23}yz+d_{24}xyz \end{array}$$

Parameter *d_i_* can be solved by the displacement of 8 nodes, then Equation (16) can be expressed as

(18) $$u=\sum_{i=1}^{8}N_{i}u_{i},v=\sum_{i=1}^{8}N_{i}v_{i},w=\sum_{i=1}^{8}N_{i}w_{i}$$

where, *N_i_* is the finite element shape function

(19) $$\begin{array}{l} N_{1}=\frac{1}{8}\left(1+\frac{x}{a}\right)\left(1-\frac{y}{b}\right)\left(1-\frac{z}{c}\right),\;N_{2}=\frac{1}{8}\left(1+\frac{x}{a}\right)\left(1+\frac{y}{b}\right)\left(1-\frac{z}{c}\right)\\ N_{3}=\frac{1}{8}\left(1-\frac{x}{a}\right)\left(1+\frac{y}{b}\right)\left(1-\frac{z}{c}\right),\;N_{4}=\frac{1}{8}\left(1-\frac{x}{a}\right)\left(1-\frac{y}{b}\right)\left(1-\frac{z}{c}\right)\\ N_{5}=\frac{1}{8}\left(1+\frac{x}{a}\right)\left(1-\frac{y}{b}\right)\left(1+\frac{z}{c}\right),\;N_{6}=\frac{1}{8}\left(1+\frac{x}{a}\right)\left(1+\frac{y}{b}\right)\left(1+\frac{z}{c}\right)\\ N_{7}=\frac{1}{8}\left(1-\frac{x}{a}\right)\left(1+\frac{y}{b}\right)\left(1+\frac{z}{c}\right),\;N_{8}=\frac{1}{8}\left(1-\frac{x}{a}\right)\left(1-\frac{y}{b}\right)\left(1+\frac{z}{c}\right) \end{array}$$

As shown in Figure 2b, assuming that the meso-model is in the macro-element, the geometric center of the macro-element is used as the origin of the coordinates to determine the coordinates of the boundary nodes of the meso-model. According to the displacement of the eight nodes of the macro-element, the displacement of each node of the meso-model boundary can be obtained after interpolation, that is, the boundary condition of the meso-model.

Since the elastic material parameters of the macro model are equivalently derived from the meso model through the homogenization period, and the boundary conditions of the meso model are interpolated from the node displacement of the macro model, the overall deformation energy of the meso model is equivalent to that of the macro model. The deformation energies of the corresponding regions of the model elements are consistent. Moreover, the boundary of the sub-model does not need to be exactly the same as the boundary of the macro-element, which greatly reduces the requirements for finite element meshing. The method proposed in this paper can be better applied to the fatigue analysis of braided composite structures.

**Figure 3:**
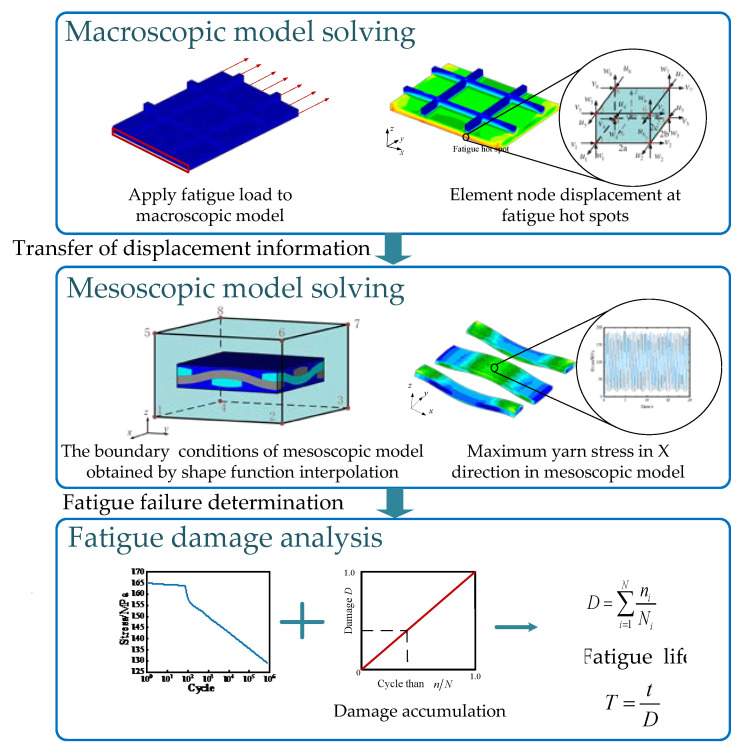
Flow chart of multi-scale fatigue analysis method for ceramic matrix composites based on dynamic sub-model.

The flow chart of multi-scale fatigue analysis method for ceramic matrix composites based on sub-model is shown in Figure 3. Firstly, establish the macro-equivalent model of CMCs structure, and apply fatigue load to obtain the stress and strain results of the macro model. Then establish the meso-cell model of CMCs. The node displacement information of the elements at the fatigue hot spots of the macro model was extracted, and the boundary node coordinates of the corresponding meso-cell model were interpolated by the shape function. The displacement information obtained after the interpolation was used as the boundary condition of the meso-cell model. Finally, the stress and strain responses of yarn and matrix are obtained by solving the meso model. The stress of the yarn was extracted, and the damage amount corresponding to each peak stress was calculated by the fatigue damage accumulation theory. After the accumulation, the damage result and residual life of the single cell model under the fatigue load could be obtained. Assuming that the structure fails at the hot spot, the structure will fail, the fatigue life of CMC structure can be obtained.

## 3. Case Study

### 3.1. Validation: Fatigue Life Calculation of 2D SiC/SiC Composites

**Figure 4:**
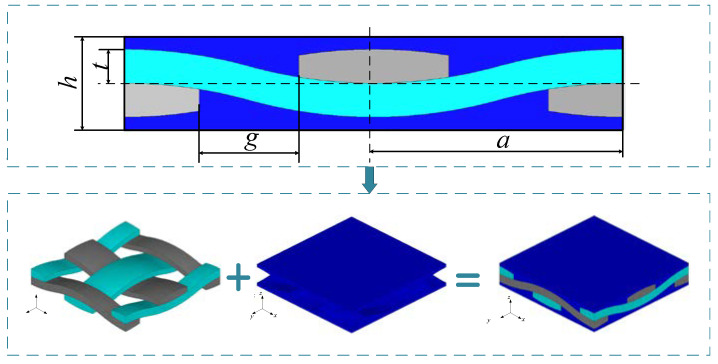
Meso-model of 2D SiC/SiC CMCs.

2D 4 SiC/SiC braided CMCs were used as the research object and analysis its fatigue life. In order to obtain the stress distribution of yarn and matrix under fatigue load, it is necessary to select a representative volume element of 2D SiC/SiC to establish a finite element model. As shown in Figure 4 it is the schematic diagram of 2D SiC/SiC meso-structure, and its mesoscopic geometric dimension parameters are shown in Table 1 [45]. The material parameters of yarn and matrix are shown in Table 2 [45]. The matrix is an isotropic material and the yarn is a transversely isotropic material. The density of the matrix is 3.22 (g/cm^3^) [46]. Think of yarn as unidirectional CMCs, the density of the yarn is selected as 2.55 (g/cm^3^) [47]. Based on the above parameters, a meso-cell finite element model of 2D SiC/SiC was established, as shown in Figure 4. The yarns along the axial direction were defined as warp and the yarns along the axial direction as weft.

For 2D SiC/SiC CMCs, it is assumed that only the effect of interface slip between fiber and matrix in yarn is considered under fatigue load, while the effect of interface slip between yarn and yarn and between yarn and matrix is ignored. Therefore, only the internal fatigue damage of the yarn is considered, and the yarn is regarded as a unidirectional ceramic matrix composite material. Firstly, the fatigue peak stress S was applied to the meso-cell sub-model. After solving the sub-model, the maximum axial stress of the yarn was extracted, which was taken into the failure determination process in Section 2.1 as the fatigue peak stress σ. Then, the number of cycle N when the yarn fatigue fails is calculated through the calculation process, which is the fatigue life of 2D SiC/SiC under the fatigue load with the peak stress. Because yarns can be considered as unidirectional CMCs, *σuts* was determined as 380 (MPa) according to Morishita’s work [48], and the data when the thickness of the interface layer is 0.1 micron is selected. The empirical parameters *P*_2_ = 1 which refers to Li’s work [49]. The empirical parameters *P*_1_ = 0.057 which is determined by fatigue ultimate stress. Other parameters are shown in Table 3 [49]

**Figure 5:**
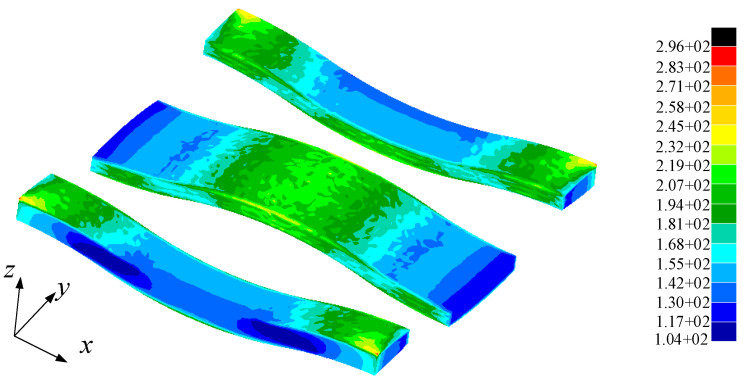
Yarn stress distribution in X direction.

One end of the 2D SiC/SiC meso-sub-model was applied with a fixed constraint and the other end with a tensile load, and the loading direction was defined as the X direction. The stress distribution of yarn in the X direction is shown in Figure 5. According to Saint-Venant principle, the region near the constraint boundary is greatly affected by the boundary condition, while the region far away from the constraint condition is less affected by the boundary condition. As can be seen from the figure, the boundary area of the outer yarn is affected by the loading mode, so there is a large local stress. The maximum stress in the other positions occurs at the lap of warp and weft, where the yarn is more bent and far away from the boundary, so the stress analysis results here can more accurately reflect the internal stress distribution of the yarn in the real situation.

**Figure 6:**
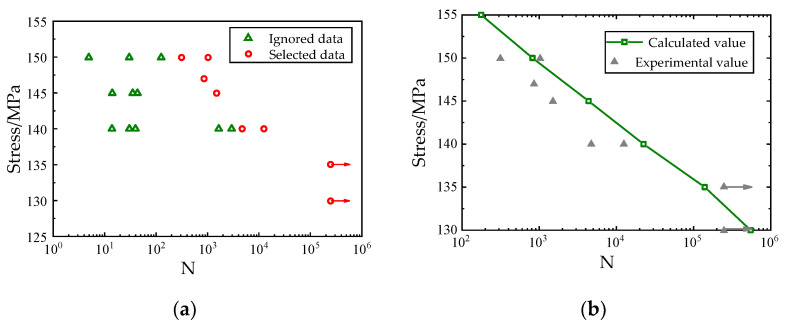
Comparison between the calculated results of fatigue life based on the submodel and the experimental results.

The meso-sub-models of 2-D SiC/SiC with peak fatigue loads of 155 MPa, 150 MPa, 145 MPa, 140 MPa, 135 MPa, and 130 MPa were calculated respectively, and the fatigue performance analysis was carried out by using the fatigue life calculation process of ceramic matrix composites based on the above sub-model, calculate the life of 2-D braided ceramic matrix composites under the corresponding stress level. Take the experimental data in [50] as reference. Due to the limitation of manufacturing process, factors such as initial defects inevitably exist in CMCs, and their fatigue performance often has greater uncertainty, so the test results in [50] have greater dispersibility. The research in this article temporarily ignores the influence of material fatigue performance uncertainty. This paper does not consider low-cycle fatigue with a stress level higher than 155 Mpa. The experimental data with better fatigue performance at fatigue load levels of 130 MPa, 135 MPa, 140 MPa, 145 MPa, 147 MPa, and 150 MPa in the reference [50] were selected, and the experimental data with poor fatigue performance due to material defects were ignored, as shown in Figure 6a. The comparison between the analysis results and the test results [49,50] is shown in Figure 6b. It can be seen from the analysis results that the fatigue analysis results based on the 2D SiC/SiC meso-sub-model are in good agreement with the test results. The accuracy of the 2D SiC/SiC meso-sub-model and the fatigue life analysis method based on the sub-mode are proved.

### 3.2. Application: Fatigue Life Analysis of Stiffened Plates

**Figure 7:**
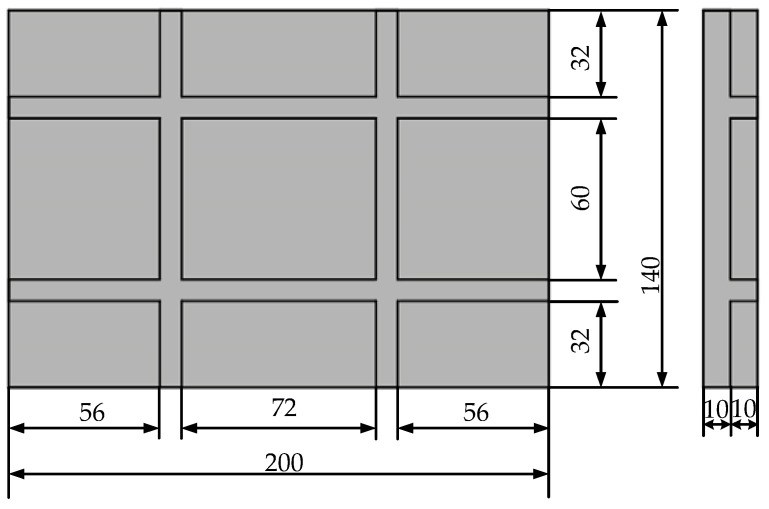
Geometric dimensions (mm) of 2D SiC/SiC stiffened plate.

In this paper, 2D SiC/SiC stiffened plate is taken as the research object, and its geometric size is shown in Figure 7. The macro-equivalent material of 2D SiC/SiC is a transversely isotropic material, and the material parameters are shown in Table 4 [45]. The axis direction is defined as the main direction of the material. The finite element model of 2D SiC/SiC stiffened plate was established. The mesh was divided by eight-node hexahedral elements with a mesh size of 4 mm. Four layers of grids were divided in the direction of the thickness of the stiffened plate and the direction of the stiffened plate. One end of the stiffened plate finite element model was subjected to a fixed constraint, and the other end to a random tension-tensile fatigue load along the X direction.

**Figure 8:**
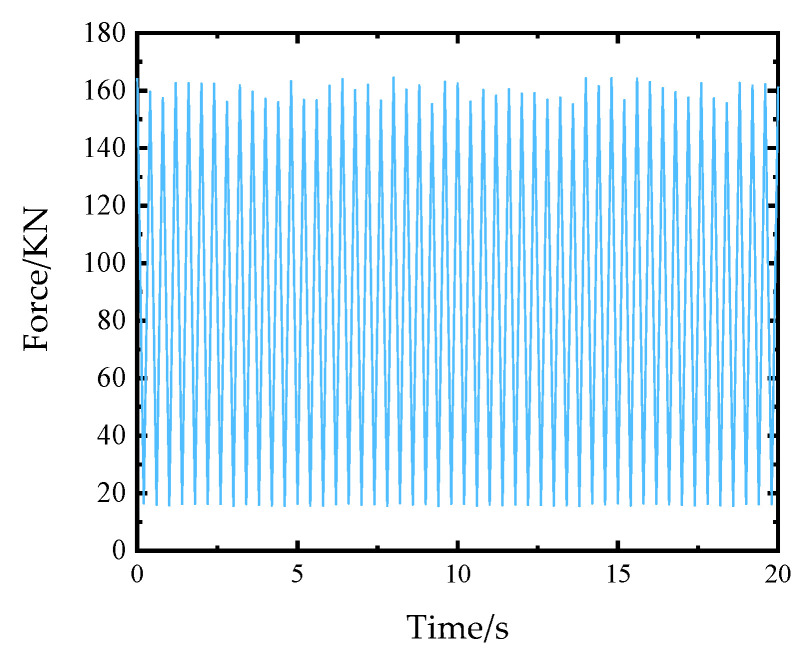
Forms of applied load.

The form and size of the load applied to the stiffened plate are shown in Figure 8. The load only retains the peak value and valley value. Peak load points are random numbers between and generated by a Gaussian stochastic process. The load unit is N, and the fatigue load stress ratio is 0.1, that is, the load valley value is 0.1 times of the corresponding peak stress. The fatigue load duration is, a total of 50 fatigue load peaks and 50 fatigue load valleys, and the loading frequency is 2.5 Hz. After applying the above load to 2D SiC/SiC stiffened plate, the finite element analysis was carried out to obtain the response results of element stress and element node displacement.

**Figure 9:**
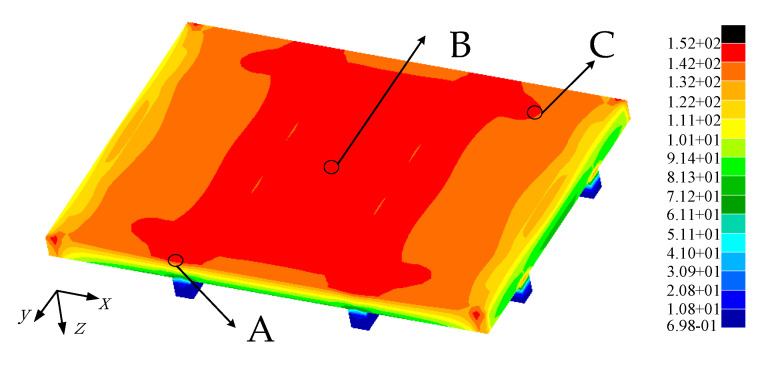
Stress in X direction distribution of 2D SiC/SiC stiffened plates under fatigue load.

**Figure 10:**
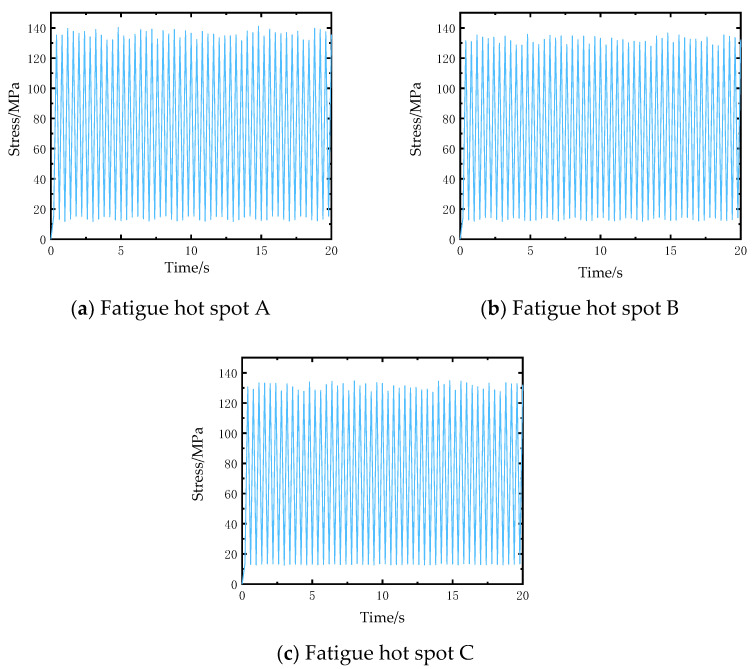
Element stress in X direction at the fatigue hot spots of the macro model.

**Figure 11:**
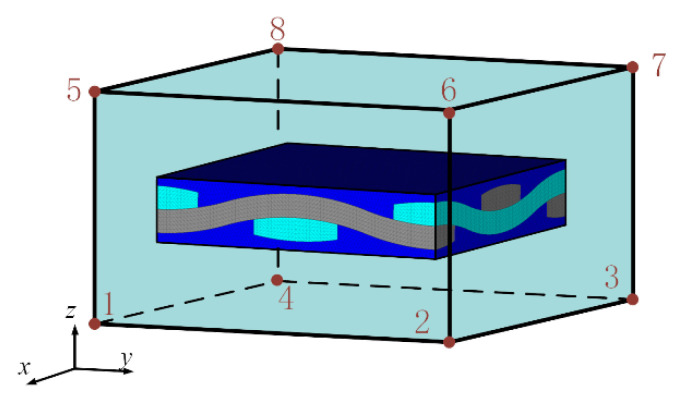
Sub-model at the center in macroscopical element.

The stress analysis results of 2D SiC/SiC stiffened plate finite element model under fatigue load is shown in Figure 9. It can be seen that under tensile load, the stress of the stiffened plate is large on the upper surface of both sides of the stiffened plate and just below the stiffened position and in the middle area of the cross stiffened plate. The high stress area of 2D SiC/SiC stiffened plate is defined as the fatigue hot spot, and three fatigue hot spots A, B, and C are selected. The elements stress results of the three fatigue hot spots are shown in Figure 10. The 2D SiC/SiC meso-cell model established in Section 3.1 was adopted as the sub-model. As shown in Figure 11, it is assumed that the geometric center of the mesoscopic single cell model coincides with the geometric center of the macroscopic element at the fatigue hotspot. The constraint conditions of each point on the boundary of the mesoscopic single cell model were obtained by the displacement response results of 8 nodes of the element at the fatigue hot spot of the macro model through interpolation in Equations (18) and (19).

**Figure 12:**
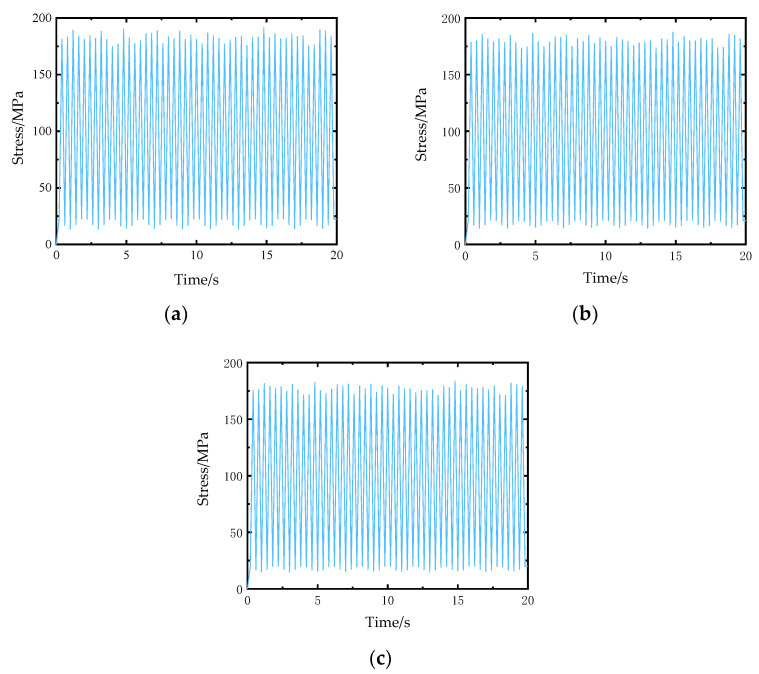
Maximum stress in X direction in yarn. (**a**) Calculation results of fatigue hot spot A; (**b**) Calculation results of fatigue hot spot B; (**c**) Cal-culation results of fatigue hot spot C.

The 2D SiC/SiC mesoscopic single cell model was analyzed by finite element method after boundary conditions were applied. According to Saint-Venant principle, the influence of boundary conditions can be ignored in the region far away from boundary constraints. According to the analysis in Section 3.1, the maximum stress in 2D SiC/SiC meso-model yarn under tensile load occurs in the yarn-to-yarn lap area, far away from the boundary point. Therefore, the maximum stress in the yarn was selected as the fatigue peak stress in the subsequent fatigue damage analysis of the yarn. The maximum stress in X direction in the yarn is shown in Figure 12.

Extract the maximum stress peak value of the schedule, yarn into 2.2 failure determination process of ceramic matrix composites, and under each peak stress calculation of the fatigue life of CMCs. Then, calculate the fatigue damage of each peak stress by Miner linear fatigue damage accumulation theory. Finally, the total fatigue damage under 20 S random fatigue load was accumulated according to Equation (14), and then the fatigue life of the cross-stiffened plate was calculated according to Equation (15). The calculated results are shown in Table 5. The analysis results show that the stress of the fatigue hotspot A is the largest, so the fatigue damage is the largest and the fatigue life is the smallest. The damage amount of hot spot B is the next, while the damage amount of hot spot C is the least and the fatigue life is the largest.

**Figure 13:**
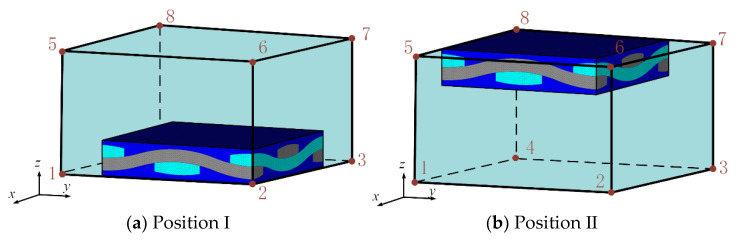
Different position of the sub-model in the unit.

**Figure 14:**
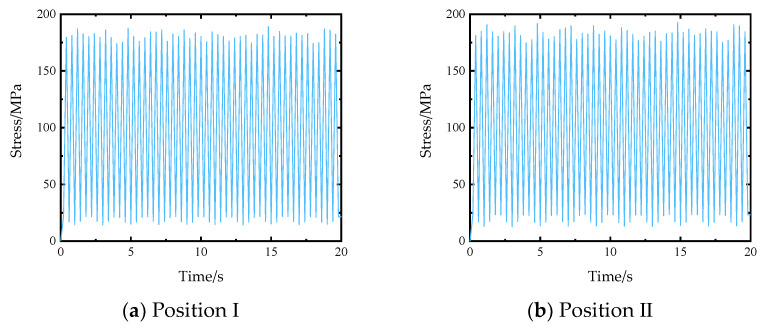
The maximum stress in the X direction of the yarn at different meso-model positions.

In actual modeling, it is difficult to ensure that the macroscopic unit size is consistent with the mesoscopic model size. When the size of the macroscopic element is larger than the size of the mesoscopic model, in order to study the influence of the position of the mesoscopic model in the macroscopic element on the calculation results, the fatigue life of the fatigue hot spot A at position I and position II as shown in Figure 13a,b is calculated respectively. When the mesoscopic model is at positions B and C, the maximum stress in the X direction of the yarn is calculated, as shown in Figure 14. The calculated fatigue damage amount and life results are shown in Table 6. Compare the analysis results of the sub-model at the center of the macroscopic element with those of other positions, it can be seen that the difference of the maximum stress in the X direction of the yarn with different sub-model position is very small, and the fatigue damage amount and fatigue life are close to each other under different positions of the macroscopic element. The results show that the positions of different mesoscopic models in the macroscopic elements have little influence on the fatigue life calculation results.

The multi-scale fatigue analysis method based on dynamic sub-models takes into account the characteristics of the mesoscopic non-uniformity caused by the complex microstructure of the braided composite material, and can more realistically reflect the fatigue damage of the structure under the fatigue load. This method has a low requirement on the modeling of macro model. When the element size of macro model is larger than the size of meso model, the position of meso model in the macroscopic element has little influence on the calculation results.

When the size of the macro unit is larger, because the shape function interpolation is a linear interpolation, the different positions of the sub-models in the macro-scale element have little effect on the calculation results. However, too large macro-scale element division will increase the calculation error of the macro-scale model itself, resulting in an increase in the calculation error of the sub-model. Therefore, when building the macro-scale model, it is necessary to minimize the size of the macro-scale element to ensure its size is close to the size of the meso-scale model. The method proposed in this paper is also applicable to the situation where the macro-scale element size is smaller than the meso-scale model size. The node displacements of multiple macro-scale elements can be used. Firstly, determine the macro-scale element where the boundary of the meso-scale model is located, and use the shape function to interpolate the displacement of the corresponding macro-element nodes, then the boundary conditions of the meso-scale model can be obtained. However, a too small macro-scale element size will reduce the calculation efficiency of the macro-scale model, and at the same time increase the difficulty of obtaining the boundary conditions of the meso-scale model.

Moreover, the multi-scale fatigue analysis method based on the dynamic sub-model can calculate the fatigue damage at different positions of the structure, which provides more references for the fatigue life design of the structure.

## 4. Conclusions

In view of the fatigue life analysis of CMCs structure, a multi-scale fatigue life analysis method based on sub-model for braided CMCs is proposed in this paper. Combining the sub-model method based on the finite element shape function and the fatigue failure criterion based on the shear lag theory, the multi-scale fatigue analysis of CMCs is implemented. Firstly, a meso-cell finite element model of 2D SiC/SiC CMCs was established, and the fatigue life of 2D SiC/SiC was analyzed by using the fatigue failure criteria of CMCs. The S–N curves were obtained and compared with the experimental results in the literature. Then, the multi-scale fatigue life analysis method based on sub-model was used to analyze the fatigue damage and life of 2D SiC/SiC cross stiffened plate under random tension–tensile loading. The fatigue damage and life of different fatigue hotspots, and different position of mesoscopic model in the macroscopic element were studied, the results show that:

(1) The fatigue life analysis results of the braided CMCs based on the sub-model are in good agreement with the experimental results in the literature, which proves the accuracy of the micro-cell finite element model of 2D SiC/SiC ceramic matrix braided composites established in this paper.
(2) The multi-scale fatigue method based on dynamic sub-model can reflect the meso-fatigue failure state of ceramic matrix composite materials, and has high computational efficiency. It can calculate the fatigue damage state of multiple positions of the structure, and the requirement for macroscopic modeling is low, only the size of the macro-scale element needs to be similar to the size of the meso-scale model, and the position of mesoscopic model in the macroscopic element has low influence on the fatigue life analysis, which is well applicable to the fatigue life analysis of two-dimensional braided CMC structures.

## Figures and Tables

**Table 1:** Meso-geometric parameters of 2D SiC/SiC CMCs.

Meso-Geometric Parameters	*t* (mm)	*g* (mm)	*a* (mm)	*h* (mm)
**Value**	0.21	0.62	1.55	0.58

**Table 2:** Material parameters of 2D SiC/SiC CMCs.

Material Parameters	Yarn	Matrix
*E*_1_(GPa)	190.01	350
*E*_2_(GPa)	190.01	350
*E*_3_(GPa)	222.31	350
*G*_12_(GPa)	64.78	145.8
*G*_13_(GPa)	79.53	145.8
*G*_23_(GPa)	79.53	145.8
*ν*_12_	0.16	0.25
*ν*_13_	0.17	0.25
*ν*_23_	0.17	0.25

**Table 3:** Relative parameters of SiC/SiC ceramic matrix braided composites.

Parameter	*τ_min_* (MPa)	*τ*(0) (MPa)	*ω*_1_	*ω*_2_	*m_f_*	*V_f_*
Value	5	50	0.04	1	2	0.4

**Table 4:** Material parameters of 2D SiC/SiC stiffened plate.

E_1_/GPa	E_2_/GPa	E_3_/GPa	G_12_/GPa	G_13_/GPa	G_23_/GPa	*ν*_12_	*ν*_13_	*ν*_23_	*ρ/*(g/cm^3^)
229.76	229.76	189.78	92.15	72.58	72.58	0.16	0.17	0.17	2.5

**Table 5:** Fatigue analysis results of 2-D braided ceramic matrix composite cross stiffened plates.

	Amount of Damage *D*	Life *T*
Fatigue hot spot A	0.000863654	23,157.42 s
Fatigue hot spot B	0.000394991	50,634.07 s
Fatigue hot spot C	0.000178935	111,772.43 s

**Table 6:** Fatigue analysis results of 2D SiC/SiC stiffened plates with different meso-model positions.

	Fatigue Damage *D*	Life *T*
Position I	0.0006534	30,607.95 s
Position II	0.00110485	18,101.96 s

## Data Availability

Not applicable.
